# Conditioned Medium from Human Tonsil-Derived Mesenchymal Stem Cells Enhances Bone Marrow Engraftment via Endothelial Cell Restoration by Pleiotrophin

**DOI:** 10.3390/cells9010221

**Published:** 2020-01-15

**Authors:** Yu-Hee Kim, Kyung-Ah Cho, Hyun-Ji Lee, Minhwa Park, Sang-Jin Shin, Joo-Won Park, So-Youn Woo, Kyung-Ha Ryu

**Affiliations:** 1 Department of Microbiology, College of Medicine, Ewha Womans University, Gangseo-Gu, Seoul 07804, Korea; kimyuhee@ewha.ac.kr (Y.-H.K.); elofan@hanmail.net (K.-A.C.); ewhalee2019@gmail.com (H.-J.L.); vin1004vin@ewha.ac.kr (M.P.); soyounwoo@ewha.ac.kr (S.-Y.W.); 2 Department of Orthopaedic Surgery, College of Medicine, Ewha Womans University, Gangseo-Gu, Seoul 07804, Korea; sjshin622@ewha.ac.kr; 3 Department of Biochemistry, College of Medicine, Ewha Womans University, Gangseo-Gu, Seoul 07804, Korea; joowon.park@ewha.ac.kr; 4 Department of Pediatrics, College of Medicine, Ewha Womans University, Gangseo-Gu, Seoul 07804, Korea

**Keywords:** tonsil mesenchymal stem cells, mesenchymal stem cell conditioned medium, bone marrow transplantation, bone marrow engraftment, pleiotrophin, vascular endothelium

## Abstract

Cotransplantation of mesenchymal stem cells (MSCs) with hematopoietic stem cells (HSCs) has been widely reported to promote HSC engraftment and enhance marrow stromal regeneration. The present study aimed to define whether MSC conditioned medium could recapitulate the effects of MSC cotransplantation. Mouse bone marrow (BM) was partially ablated by the administration of a busulfan and cyclophosphamide (Bu–Cy)-conditioning regimen in BALB/c recipient mice. BM cells (BMCs) isolated from C57BL/6 mice were transplanted via tail vein with or without tonsil-derived MSC conditioned medium (T-MSC CM). Histological analysis of femurs showed increased BM cellularity when T-MSC CM or recombinant human pleiotrophin (rhPTN), a cytokine readily secreted from T-MSCs with a function in hematopoiesis, was injected with BMCs. Microstructural impairment in mesenteric and BM arteriole endothelial cells (ECs) were observed after treatment with Bu–Cy-conditioning regimen; however, T-MSC CM or rhPTN treatment restored the defects. These effects by T-MSC CM were disrupted in the presence of an anti-PTN antibody, indicating that PTN is a key mediator of EC restoration and enhanced BM engraftment. In conclusion, T-MSC CM administration enhances BM engraftment, in part by restoring vasculature via PTN production. These findings highlight the potential therapeutic relevance of T-MSC CM for increasing HSC transplantation efficacy.

## 1. Introduction

Hematopoietic stem cell transplantation (HSCT) is a definitive treatment option for incurable hematologic malignances including leukemia, multiple myeloma, and aplastic anemia. HSCs administered into circulating peripheral blood migrate to and engraft in bone marrow (BM), which is preconditioned with high doses of cytotoxic drugs and irradiation. Until the transplanted HSCs engraft and recover myeloid lineages of blood cells, intensive care is required to prevent hemorrhage and infection. A retrospective study revealed that primary graft failure occurs in 2.5 to 7.3% of cases [1]. Therefore, enhancing BM engraftment could be a useful strategy for increasing HSCT treatment efficacy.

Studies have offered strategies to enhance BM engraftment by promoting the interaction between homing chemokine (stromal cell-derived factor 1, SDF-1) and CXC chemokine receptor type 4 (CXCR4) [2]. Stabilization of CXCR4 on membrane lipid -rafts of HSCs [3] and/or upregulation of CXCR4 expression by pretreatment with platelet-derived extracellular microvesicles have been suggested [4]. Chemical or physical modulation of HSCs ex vivo prior to transplantation has also been proposed to improve engraftment and survival [5,6,7,8].

Cotransplantation of autologous or allogeneic mesenchymal stem cells (MSCs) with HSCs promoted BM engraftment in several clinical studies [9,10,11]. MSC transplantation was also deemed effective for patients developing graft failure and poor graft function [12,13]. Studies suggest that the therapeutic effects of MSCs in HSC transplantation involve reconstitution of damaged BM stroma, secretion of hematopoietic cytokines, and modulation of immune responses [14].

Previously, we demonstrated that human palatine tonsil-derived MSCs (T-MSCs) show tissue regenerative and immunomodulatory effects [15,16,17]. In addition, T-MSC infusion in allogeneic BM transplantation (BMT) and senile osteoporosis mouse models improved BM reconstitution [18,19]. In the BMT mouse model, cotransplantation of T-MSCs with BMCs enhanced myelopoiesis with little effect on erythropoiesis. In senile osteoporosis, where diminished hematopoietic structure and function is present, T-MSC transplantation or conditioned medium (CM) was efficient to increase BM cellularity.

In this study, we aimed to identify the effectiveness of T-MSC CM in promoting BM engraftment using an allogeneic BMT mouse model. We hypothesized that T-MSC secretory factors could recapitulate the beneficial effects of MSC cotransplantation. To address this, we injected T-MSC CM into BMT mice to examine bone marrow reconstitution and the effects on mesenteric and BM endothelial cells (ECs).

## 2. Materials and Methods

### 2.1. Allogeneic Bone Marrow Transplantation

Recipient 8-week-old female BALB/c mice (H-2^d^) and donor male C57BL/6 mice (H-2^b^) were purchased from Orient Bio (Sungnam, South Korea). Mice were housed at 21–23 °C and 51%–54% humidity with a 12-h light/dark cycle under conventional conditions, and food and water were supplied ad libitum. BM ablation was induced by administration of busulfan (Bu, 20 mg/kg) for 4 days followed by cyclophosphamide (Cy, 100 mg/kg) for 2 days. After a day of rest, preconditioned mice were randomly divided into five groups, as follows: Group 1, Bu–Cy chemotherapy (Bu–Cy); Group 2, bone marrow transplantation (BMT); Group 3, BMT with T-MSC CM injection (BMT + CM; 200 μL, derived from 10^6^ T-MSCs); Group 4, BMT with recombinant human PTN (rhPTN, 50 μg/kg, 252-PL, R&D Systems, Minneapolis, MN, USA) treatment (BMT + rhPTN); Group 5, BMT with T-MSC CM and anti-PTN neutralizing antibody (α-PTN Ab, 250 μg/kg, AF-252, R&D Systems) treatment (BMT + CM + α-PTN Ab). Donor BMCs were isolated from C57BL/6 mouse lower limbs (femurs and tibias) and 1.5 × 10^6^ cells were resuspended in 200 μL of DMEM or CM and transplanted to recipient mice via tail vein. CM, rhPTN, or CM + α-PTN Ab was injected 2 days later. Mice were sacrificed on days 4, 10, or 21 after BMT for analysis. Experiments and procedures were approved by the Animal Ethics Committee at Ewha Womans University School of Medicine (ESM 16-0354), and all experiments were performed in accordance with relevant guidelines and regulations, including institutional animal care and use committee (IACUC) protocol approval.

### 2.2. T-MSC Culture and Preparation of CM

T-MSCs were isolated from human palatine tonsils obtained from patients undergoing tonsillectomy at Ewha Womans University Mok-Dong Hospital (Seoul, South Korea; approved by the Institutional Review Board; no. EUMC 2018-01-011-002), and were cultured in DMEM high glucose medium (Welgene, Gyeongsan, South Korea) supplemented with 10% FBS, 100 IU/mL penicillin, and 100 μg/mL streptomycin (Welgene) [20,21]. Culture media was changed every 3–4 days. To collect CM, T-MSCs were cultured to 80% confluency. After replacing the culture medium with fresh serum-free medium, cells were incubated for an additional 48 h and CM was harvested. CM was concentrated using a 3-kDa Amicon Ultra centrifugal filter unit (EMD Millipore, Darmstadt, Germany) by centrifugation at 4500 rpm at 4 °C for 1 h.

### 2.3. Cell Labeling with PKH26

BMCs were labeled with the PKH26 red fluorescent cell linker kit (Sigma-Aldrich, St. Louis, MO, USA) according to the manufacturer’s instructions. For BM homing of PKH26-labeled cells, femurs were collected from mice on day 4 after BMT, flushing BMCs out with a syringe; then PKH26-positive cells were analyzed on a NovoCyte flow cytometer (ACEA Biosciences, San Diego, CA, USA) and analyzed using NovoExpress software (ACEA Biosciences).

### 2.4. Hematological Analysis

On the day of sacrifice, mice were anesthetized using isoflurane (Hana Pharm, Seoul, South Korea) and blood was collected by cardiac puncture. Blood samples were diluted 100-fold in PBS containing 10% FBS, 10 mM EDTA, and 20 mM HEPES, and further diluted 1000-fold in Isoton II Diluent (Beckman Coulter, Indianapolis, IN, USA). Numbers of red blood cells (RBCs) and white blood cells (WBCs) were counted using a Coulter counter fitted with a cell-sizing analyzer (Beckman Coulter).

### 2.5. Hematoxylin & Eosin (H&E) Staining

Mouse femurs were isolated and fixed with 10% formalin solution in PBS. Decalcified and paraffin-embedded femurs were sectioned at a 4-μm thickness and subjected to H&E staining for histological evaluation. Stained sections were scanned with an Aperio ScanScope slide scanner (Leica Biosystems, Wetzlar, Germany) and analyzed using CaseViewer software (3Dhistech, Budapest, Hungary). Images were captured under 100× magnification. Bone marrow cellularity was calculated from more than eight different fields using NIH ImageJ 1.52a software (https://imagej.nih.gov/).

### 2.6. Immunohistochemistry

Sections of paraffin-embedded femurs were hydrated and subjected to heat-induced epitope retrieval in citrated buffer (10 mM citrate containing 0.05% Tween 20, pH 6.0) for 20 min at 95 °C. After cooling down the slides, protein blocking was performed using a Dako universal blocking solution (Agilent, Santa Clara, CA, USA) for 15 min in a humidity chamber. Specimens were incubated with a primary antibody against mouse endomucin (V.7C7, sc-65495; Santa Cruz Biotechnology, Santa Cruz, CA, USA) overnight at 4 °C, then with a rat AP polymer (Abcam) for 30 min at room temperature. Immunoreactivity was developed using Permanent Red Chromogen (Abcam) for 10 min, observing appropriate color development. Hematoxylin counterstain was performed and visualized using a ScanScope slide scanner, with images captured at 200× and 400× magnifications. The percentage of endomucin-stained areas in femur endosteal regions was determined using ImageJ software.

### 2.7. Flow Cytometry

To perform flow cytometry analysis of circulating mononuclear cells, blood collected from cardiac puncture was immediately mixed in FACS buffer (PBS supplemented with 10% FBS, 10 mM EDTA, 20 mM HEPES, and 1 mM sodium pyruvate). Cells were pelleted by centrifugation at 1300 rpm for 5 min, and RBS lysis was performed by incubation in ACK lysis buffer (150 mM NH_4_Cl, 10 mM KHCO_3_, 0.1 mM Na_2_ EDTA). After washing the cells twice with FACS buffer, RBC lysis was repeated if required. To determine MHC haplotype, cells were stained with FITC-conjugated H-2^b^ (SF1-1.1, mouse IgG2a; BD Biosciences, San Jose, CA, USA) and PE-conjugated anti-mouse H-2^d^ (AF6-88.5, mouse IgG2a; BD Biosciences) antibodies. Circulating endothelial cells were examined by staining with PE-conjugated anti-mouse CD45 (30-F11, rat IgG2b; BD Biosciences) and Alexa Fluor 647 anti-mouse CD144 (BV13, rat IgG1; Biolegend, San Diego, CA, USA) antibodies. After washing cells with buffer, protein expression was measured on a NovoCyte flow cytometer and analyzed using NovoExpress software.

### 2.8. Transmission Electron Microscopy (TEM)

Mesenteric vessels were harvested from intestinal arteries of the second and third branching order and immediately perfused with 2.5% glutaraldehyde in 0.1 M phosphate buffer (pH 7.4). Specimens were washed in 0.1 M phosphate buffer and post-fixed with 1% osmium tetroxide in 0.1 M phosphate buffer (pH 7.4) for 1 h, dehydrated with ethanol, and embedded in epoxy-resin. Semi-thin sections were prepared at a 1-μm thickness and stained with toluidine blue. Ultrathin sections, approximately 60–70 nm in thickness, were cut by an ultramicrotome (Leica EMUC7) using a diamond knife. Sections were contrasted with uranyl acetate followed by lead citrate and observed with H-7650 TEM (Hitachi, Tokyo, Japan) at an accelerating voltage of 80 kV.

### 2.9. HUVEC Culture and MTT Assay

Human umbilical vein endothelial cells (HUVECs; CRL-1730) were purchased from American Type Culture Collection (ATCC, Manassas, VA, USA) and cultured in Ham’s F-12K medium (Welgene) supplemented with Large Vessel Endothelial Supplement (Thermo Fisher Scientific, Waltham, MA, USA). Cells were seeded into 96-well culture plates (0.5 × 10^4^ cells/well), and while in proliferative stages, were treated with Bu or Cy for 4 h to induce cytotoxic effects followed by CM (secreted from 0.5 × 10^4^ T-MSCs), rhPTN (200 ng/mL), or CM + α-PTN Ab (1 μg/mL) treatment for 16 h. To determine the number of live cells, thiazolyl blue tetrazolium bromide (MTT) was reconstituted in PBS (5 mg/mL), then 20 μL was added to each well. After 4 h of incubation, culture supernatant was removed and 200 μL DMSO was added to elute the formazan production for absorbance measurement at 570 nm.

### 2.10. Tube Formation Assay

A 96-well plate was coated with Matrigel Matrix (BD Biosciences) for 1 h at room temperature. After washing with PBS, HUVECs pretreated with Bu or Cy for 4 h were seeded into a pre-coated plate (2 × 10^4^ cells/well) and incubated in a CO_2_ incubator with or without CM, rhPTN, or CM + α-PTN Ab. The next day, tube formation was photographed and analyzed using ImageJ software in conjunction with Angiogenesis Analyzer.

### 2.11. Western Blot

After washing cells twice with ice-cold PBS, cells were incubated on ice for 15 min in protein lysis buffer containing 20 mM HEPES, 1% Triton X-100, 150 mM NaCl, 1 mM EDTA, 2 mM Na_3_VO_4_, 10 mM NaF, and protease inhibitor cocktail (P8340, Sigma-Aldrich). Cells were harvested using a cell scraper, and cell lysates were prepared by centrifugation at 13,000 rpm for 15 min at 4 °C. Supernatant was collected and protein concentrations were determined by Pierce BCA Protein Assay kit (Thermo Fisher Scientific). Samples were resolved by SDS-PAGE and transferred to PVDF membrane (EMD Millipore). After blocking membranes in 5% skim milk containing TBS-T (*w*/*v*) for 1 h at room temperature, membranes were incubated in primary antibodies (PTN, 1:500 dilution in 2% BSA containing TBS-T, sc-74443, Santa Cruz and β-actin, 1:2000 dilution in 2% BSA containing TBS-T, A1978, Sigma-Aldrich) overnight at 4 °C. Membranes were washed three times for 10 min in TBS-T and incubated in anti-mouse HRP-conjugated secondary antibody (1:3000 dilution in TBS-T, Bio-Rad, Hercules, CA, USA) for 1 h at room temperature. Following washing, membranes were developed using SuperSignal West Femto Substrate (Thermo Fisher Scientific) and scanned using ImageQuant LAS 4000 (GE Healthcare, Little Chalfont, UK).

### 2.12. ELISA

Secreted levels of hPTN in AT-, BM-, or T-MSC CM were quantified using a human PTN ELISA kit (RayBiotech, Norcross, GA, USA) according to the manufacturer’s instructions. CM harvested from 106 cells were used for the analysis.

### 2.13. Statistical Analysis

Statistical analyses were performed using one-way ANOVA in conjunction with Tukey’s post-hoc test to compare differences between the respective treatment groups in GraphPad Prism 7.03 (GraphPad Software, La Jolla, CA, USA). Data are presented as the mean ± S.E.M. *P*-values < 0.05 were considered to be statistically significant.

## 3. Results

### 3.1. T-MSC CM Treatment Enhances Survival by Promoting BM Reconstitution after BMT

**Figure 1:**
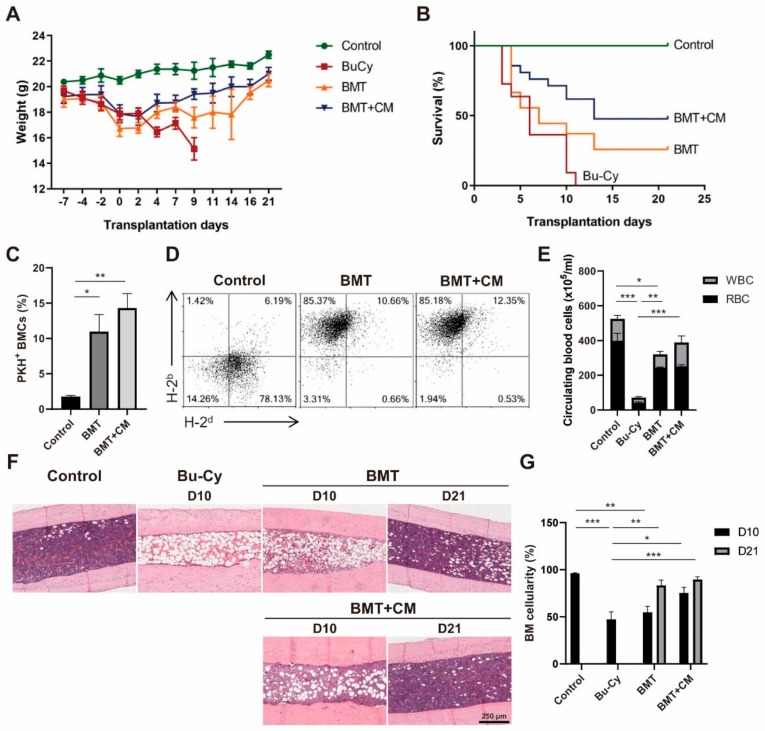
T-MSC CM treatment promotes BM reconstitution after BMT. BMT was performed in mice preconditioned with Bu–Cy administration with or without T-MSC CM. (**A**) Mouse body weight was measured throughout the course of experiments. (**B**) Survival rate was determined using Kaplan–Meier estimates. (**C**) PKH26-labeled donor cells were detected in mouse femurs on day 4 post-BMT using flow cytometry. (**D**) Analyses of MHC haplotype expression of blood mononuclear cells were conducted on day 10 post-BMT using flow cytometry. H-2^b^ represents the donor C57BL/6 cells and H-2^d^ for BALB/c recipient. (**E**) The numbers of RBCs and WBCs in peripheral blood were examined using a Coulter counter cell-sizing analyzer on day 10 post-BMT. (**F**) Histological analysis of BM cellularity was performed on day 10 and 21 post-BMT, and representative images of H&E staining from mouse femurs are shown (100× magnification). (**G**) BM cellularity was measured from more than eight different fields on days 10 and 21 using ImageJ software. Data are presented as mean ± S.E.M. and were analyzed using one-way ANOVA (*n* = 12, * *p* < 0.05, ** *p* < 0.01, *** *p* < 0.001). T-MSC CM, tonsil-derived mesenchymal stem cell conditioned medium; BM, bone marrow; BMT, bone marrow transplant; Bu–Cy, busulfan and cyclophosphamide; RBC, red blood cells; WBC, white blood cells.

In our previous study, we demonstrated that cotransplantation of T-MSCs improved BMT efficacy in an allogeneic BMT mouse model [18]. We extended these studies here by investigating the paracrine effects of MSCs in cotransplantation using T-MSC CM. We examined weight changes following BM ablation and BMT. Results showed that Bu–Cy preconditioning induced a sharp decrease in mouse body weight sufficient enough to indicate sacrifice by day 10 post-BM ablation in accordance with humane endpoints. BMT protected mice from further weight loss, and CM addition appeared to enhance weight gain (Figure 1A). Percent survival of each group of mice is illustrated in Figure 1B. The survival rate increased from 25.9% to 47.6% for BMT supplemented with CM. Median survival also increased from 7 days to 13 days. In order to determine a homing efficiency of donor BMCs, PKH26-labelled BMCs were transplanted and harvested from femurs. The homing efficiency increased from 10.97% to 14.31% on day 4 post-BMT (Figure 1C). On day 10 post-BMT, mononuclear cells were isolated from peripheral blood to examine the expression of MHC haplotypes. Results demonstrated a replacement of H-2^d^-expressing blood cells to H-2^b^, suggesting that BM reconstitution was achieved in BMT recipients (Figure 1D). The number of cells in circulation on day 10 post-BMT was partially recovered in the BMT group, while CM treatment enhanced blood cell recovery to levels similar to the control group (Figure 1E). To identify the effects of CM treatment through BM histological changes, we performed H&E staining using paraffin-embedded femur samples. Changes in BM cellularity were measured on days 10 and 21 post-BMT (Figure 1F). Interestingly, a significant increase in BM cellularity was observed in the CM treatment group by day 10 compared to BMT alone. By day 21, BM reconstitution was complete in post-BMT mice with similar degrees of BM cellularity between BMT and BMT + CM treatment groups (Figure 1G). These results suggest that CM treatment promotes and accelerates BM engraftment resulting in enhanced mouse survival after BMT.

### 3.2. PTN Secreted from T-MSCs Promotes BM Engraftment

**Figure 2:**
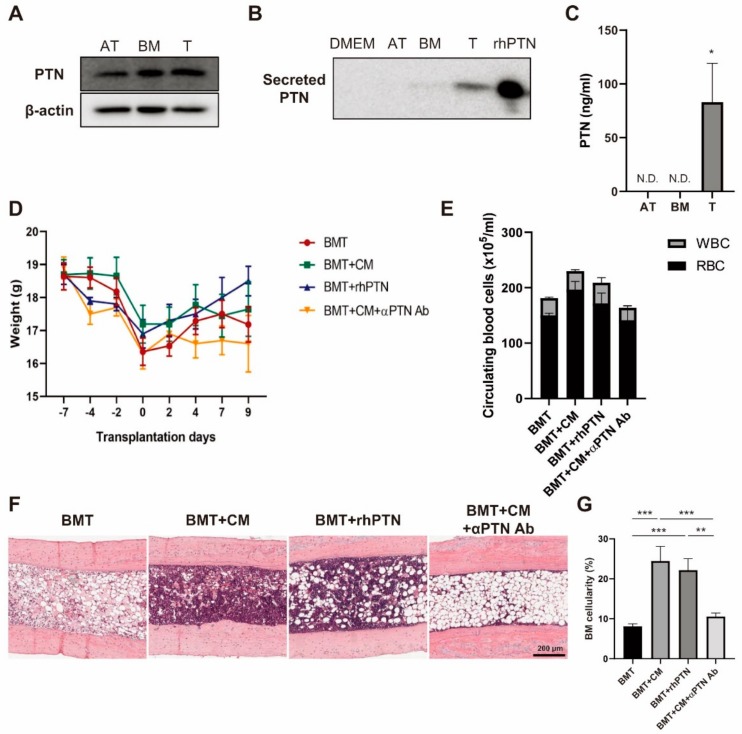
T-MSCs produce pleiotrophin (PTN) and promote BM engraftment. PTN expression levels in (**A**) whole-cell lysates and (**B**) conditioned media of BM-, AT-, or T-MSCs were determined by western blot; 1 ng of rhPTN was loaded in parallel. (**C**) Secreted levels of PTN in CM of BM-, AT-, or T-MSCs were quantified by ELISA. (**D**) BMT was performed in the presence of CM, rhPTN, or CM + anti-PTN antibody, and mice were sacrificed on day 10 post-BMT (*n* = 5). Body weight changes are indicated. (**E**) The number of circulating RBC and WBC were counted. (**F**) Histological BM changes were determined by H&E staining of mouse femurs (100× magnification) and (**G**) BM cellularity was measured from more than eight different fields using ImageJ software. Data are presented as mean ± S.E.M. and were analyzed using one-way ANOVA (** *p* < 0.01, *** *p* < 0.001).

Previously, we performed a transcriptome sequencing analysis of MSCs derived from BM, adipose tissue (AT), and tonsil [21]. We listed genes that are highly upregulated in T-MSCs compared to AT-MSCs, but show similar expression levels to BM-MSCs, in order to find out a novel regulator expressed in T-MSCs that may play roles in BM regeneration. It was revealed that PTN, a key player in the maintenance of hematopoiesis [22,23], is highly expressed in T-MSCs compared to AT-MSCs. We next investigated the role of PTN secreted from T-MSCs in BM engraftment. PTN protein expression levels were found to be higher in BM- and T-MSCs as compared to AT-MSCs (Figure 2A). We also examined secretion of PTN protein into culture media by western blot and found that T-MSCs readily secrete PTN compared to BM- or AT-MSCs (Figure 2B). Quantitation of PTN secretion using ELISA also showed that T-MSCs secrete 83.05 ± 25.53 ng/mL PTN while in CM of AT- or BM-MSCs was under the detection limits (Figure 2C).

Next, we investigated the effects of PTN treatment on BM engraftment using the BMT mouse model. Bu–Cy preconditioned mice were divided into four groups, and BMT was performed with supplementation by T-MSC CM, rhPTN, or CM with anti-PTN blocking Ab. Given that CM treatment accelerated BM reconstitution by day 10, we chose day 10 to sacrifice the mice post-BMT for analysis. There were no significant difference in body weight between groups, although the CM and rhPTN supplemented groups showed slightly higher body weights than the BMT or CM + anti-PTN Ab supplemented groups (Figure 2D). The number of circulating blood cells significantly increased in the CM-treated group compared to BMT and CM + anti-PTN Ab treatment groups (Figure 2E). BM cellularity determined by H&E staining demonstrated that CM and rhPTN treatments significantly increased BM cellularity compared to the untreated BMT group (Figure 2F,G). PTN likely promotes BM reconstitution in CM treatment, as BM engraftment was delayed in CM + anti-PTN Ab mice.

### 3.3. PTN within T-MSC CM Restores Mesenteric Endothelium

**Figure 3:**
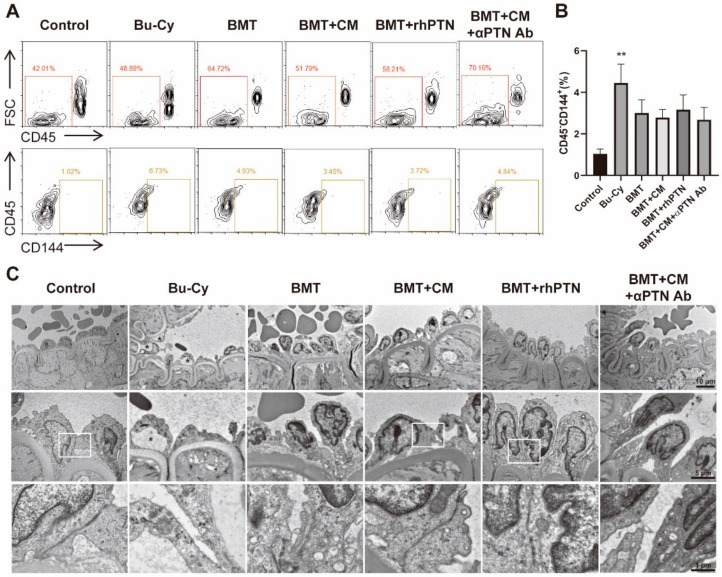
T-MSC CM treatment restores mesenteric endothelium. Mice were sacrificed on day 4 post-BMT and blood mononuclear cells and mesenteric vessels were harvested for analyses (*n* = 5). (**A**) Circulating endothelial cells (ECs; CD45^−^CD144^+^) were determined using flow cytometry, and representative images are shown. (**B**) Percentage of circulating ECs in each experimental group was determined. (**C**) Representative images of transmission electron microscopy in each experimental group (5000×, 10,000×, and 30,000× magnifications from the top). Mesenteric artery of control group shows a uniform endothelial surface with well-constructed intercellular junctions (arrow). Bu–Cy group shows a desquamation in artery endothelial cells and generation of large vacuoles (asterisk) between endothelial cells and elastin lamina as well as between elastin lamina and smooth muscle cells. Data are presented as mean ± S.E.M. and were analyzed using one-way ANOVA (* *p* < 0.05, ** *p* < 0.01, *** *p* < 0.001).

Increased ECs in circulation is an indicator of EC injury after treatment with cytotoxic drugs like Bu and Cy [24,25]. In order to determine if BMT and CM treatment could restore the injured ECs, we examined circulating EC levels (CD45^-^CD144^+^) using flow cytometry on day 4 post-BMT. As expected, Bu–Cy treatment induced mobilization of ECs to circulation, while BMT slightly reduced the levels of circulating ECs. CM or rhPTN supplementation did not offer any significant additive effects to BMT on reducing circulating EC levels (Figure 3A,B). Next, we examined the microstructure of the mesenteric endothelium (Figure 3C). Mesenteric endothelium of control mice showed a uniform endothelial surface with well-structured interendothelial junctions. Bu–Cy treatment induced EC injury detected by cytoplasmic vacuolation and retraction of ECs. Disruption of cell-to-cell contacts leading to gaps between adjacent ECs was evident. In addition, a high-magnification view revealed a loss of cell organelles and mitochondrial swelling. After BMT, the endothelial lining seemed partially recovered. However, ECs showed features of inflammation as demonstrated by peripheralization of chromatin and nucleoli, protrusion into the capillary lumen, and dispersed small vacuoles throughout the cytoplasm. CM or rhPTN treatment supplementation to BMT promoted the recovery of mesenteric arterial ECs as compared to the BMT group, in addition to an increased frequency in the formation of intercellular junctions. The microstructure of mesenteric ECs in the CM + anti-PTN Ab group showed a similar morphology to BMT alone, suggesting that PTN plays a critical role in the restoration of mesenteric endothelium after BMT.

### 3.4. T-MSC Produce PTN to Restore BM Arteriole Endothelium

**Figure 4:**
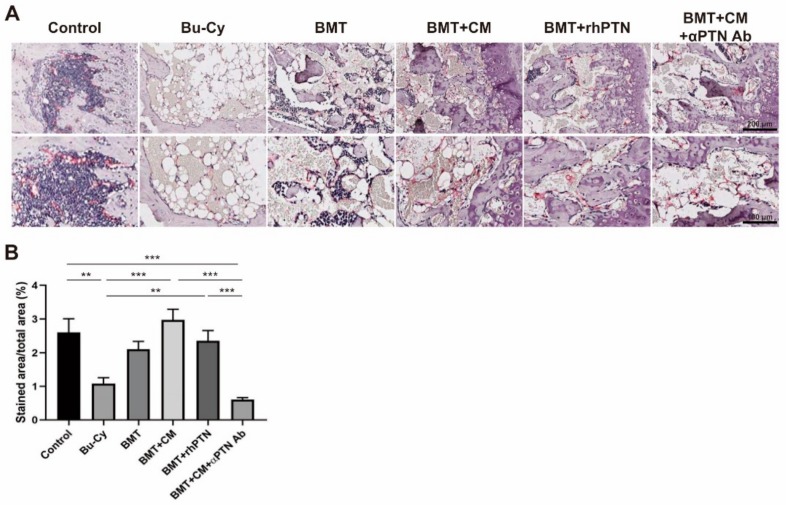
T-MSC CM treatment restores BM endothelium. (**A**) Expression of endomucin was determined by immunohistochemistry from mouse femurs on day 4 post-BMT, and representative images are shown (200× magnification in upper panel; 400× magnification in lower panel). (**B**) Percentage of endomucin-stained areas per total area was measured from more than eight different fields using ImageJ software. Data are presented as mean ± S.E.M. and were analyzed using one-way ANOVA (** *p* < 0.01, *** *p* < 0.001).

Given that BM ECs play an important role for HSC maintenance within the BM [26,27], we next performed a histological analysis of BM ECs using an endomucin antibody. Endomucin is a selective marker for small arterioles in the BM endosteal region where transplanted long-term, repopulating HSCs reside [28,29]. Endomucin, highly expressed in control mouse femurs, was reduced in Bu–Cy-treated mice while BMT slightly restored this expression. CM treatment significantly improved endomucin expression, and rhPTN showed similar effects to CM treatment. These regenerative effects of CM and rhPTN were disrupted by the presence of the anti-PTN Ab (Figure 4A,B).

### 3.5. PTN Secreted from T-MSCs Recovers Damaged HUVECs

**Figure 5:**
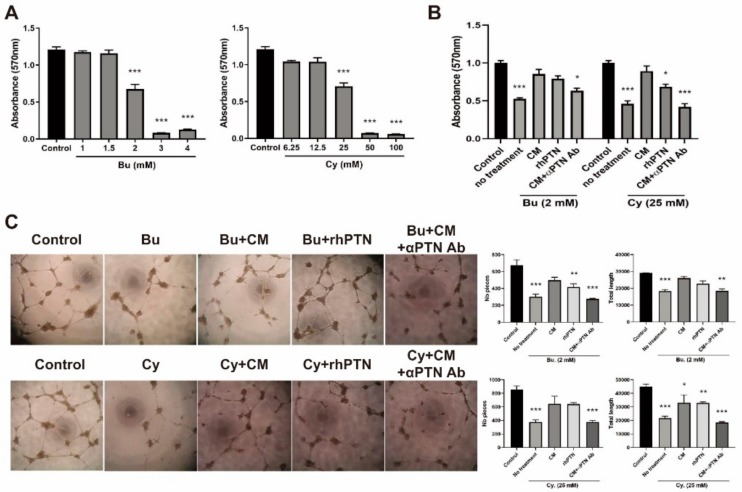
T-MSC CM restores cytotoxic drug-injured HUVECs by secreting PTN. (**A**) Cytotoxicity of Bu or Cy on HUVECs at various concentrations was examined using MTT assay. (**B**) Effects of CM or rhPTN on cell viability of damaged HUVECs were determined by MTT assay. (**C**) Tube formation assay was performed using damaged HUVECs followed by CM or rhPTN treatment. Total number of pieces (segments, isolated elements, and branches) and total lengths were used for the quantitation of the tube formation. Data are presented as mean ± S.E.M. and were analyzed using one-way ANOVA (*n* = 3, * *p* < 0.05, ** *p* < 0.01, *** *p* < 0.001).

The effects of T-MSC CM and rhPTN on damaged ECs were determined in vitro using HUVECs. First, cells were challenged with various concentrations of Bu or Cy followed by MTT assay to examine cell viability and determine the LC_50_ concentration (Figure 5A). Next, HUVECs were treated with this defined concentration of Bu or Cy for 4 h followed by CM or rhPTN treatment. CM and rhPTN protected cells from drug-mediated cell death, and these pro-survival effects were abolished in the presence of the anti-PTN antibody (Figure 5B). Next, the tube formation of damaged ECs was investigated on Matrigel-coated plastic dishes. Cells pretreated with Bu or Cy showed defects in vascular morphogenesis compared to normal HUVECs as determined by counting the total number of segments and branches and total length. Tube formation was restored in CM and rhPTN treatment groups, whereas the anti-PTN antibody inhibited the effects of CM (Figure 5C). These results support the notion that CM and rhPTN may accelerate BM engraftment through the restoration of ECs.

## 4. Discussion

In the current study, we demonstrated that T-MSC CM accelerates BM engraftment and EC recovery in a mouse model of allogeneic BMT. Cytotoxic drugs were used to induce BM ablation in mice, and BMT was conducted in the presence or absence of T-MSC CM. Our data suggest that T-MSC CM supports BM engraftment to increase mouse survival. We also showed that PTN is expressed in T-MSCs at similar levels as BM-MSCs but is more readily secreted from T-MSCs. For promoting BM cellularity and BM EC recovery, recombinant PTN treatment showed similar, but lesser, effects to T-MSC CM treatment. Thus, T-MSC CM efficiently promotes BM reconstitution, which could be considered a novel therapeutic strategy to enhance HSCT efficacy.

HSCT is considered the best treatment option for curing hematologic malignancies. However, this therapy involves high risks of infection, hemorrhage, graft failure, graft-versus-host disease, and relapse. Therefore, the development of a supplementary remedy to accelerate HSC homing and BM engraftment is required for enhancing clinical outcomes after HSCT. Prior strategies include modulation of HSC responsiveness to BM homing molecules using chemical or physical treatment [2]. In this report, we have shown that treatment with T-MSC CM could promote BM engraftment and EC recovery, supporting the use of MSC CM as a novel therapeutic strategy to protect patients from early graft failure and reduce the period of BM function nadir.

MSC and HSC cotransplantation enhanced the treatment outcome in several clinical and preclinical studies of HSCT [9,10,11,18,30]. The immunomodulatory effects of MSCs and production of paracrine factors are proposed mechanisms for supporting BM engraftment. MSCs produce cytokines and growth factors that contribute to recovery from injury by promoting cell proliferation, inhibiting apoptosis, regulating immune responses, and activating endogenous stem cells. Our research group has been using human palatine tonsil as a novel supply of MSCs, and the therapeutic effects of T-MSCs have been investigated in various mouse models of disease including psoriasis [17], BM ablation [18], osteoporosis [19], and liver cirrhosis [31]. Here, we demonstrate that T-MSC paracrine factors enhance BMT effectiveness and CM treatment alone could elicit these effects without the cells.

The use of MSC CM provides several advantages over cell-based therapy while preserving the therapeutic effects [32,33]. First of all, use of CM is often considered to be safer than cell-based therapy since living and proliferative cell transplantation involves safety issues regarding immune compatibility and tumorigenicity. In addition, CM is easier for manufacturing, storage, and handling so that off-the-shelf therapies could be possible economically and practically. Preconditioning of MSCs can be considered with the aim of modifying profiles of secretome. By inducing a hypoxic condition, culturing cells in tri-dimension, or providing a mechanical stiffness, MSC secretome could be modulated [32,34]. Significant increases in hepatocyte growth factor and vascular endothelial growth factor secretion in T-MSCs cultured under hypoxia was observed (data not shown). Therefore, efforts should be made for further enhancing preclinical outcomes of BMT.

Human palatine tonsils are considered as a novel MSC source that possess several advantages over other tissue sources [35,36]. Above all things, MSC yield and doubling time is superior to BM-MSCs. Previously, we performed transcriptome sequencing analysis of BM-, AT-, and T-MSCs and reported on the distinguishing characteristics of T-MSCs. Through functional enrichment analysis, we found that T-MSCs highly express genes involved in cell adhesion, extracellular matrix (ECM) remodeling, cell proliferation, and angiogenesis compared to BM- or AT-MSCs [21]. BM engraftment includes the need for transplanted BMCs to migrate through blood vessels via cell adhesion molecule anchoring and home to the BM niche, following gradient changes of chemoattractants [37]. In addition, BM engraftment involves the enzymatic processing of ECM components that surround the niche [38,39] followed by the proliferation of HSCs. T-MSC paracrine factors could play roles in the steps of BM engraftment, so identifying the specific effector molecules would provide a framework for developing novel therapeutics. Here, we offer PTN as a candidate molecule that promotes BM engraftment via regeneration of BM ECs. The function of PTN in HSC maintenance and regeneration is well-characterized [22,23]. More recently, Himburg and colleagues have reported that PTN dichotomously regulates hematopoiesis under steady-state and injury [40]. It was shown that PTN expressed from BM perivascular stromal cells plays a role in homeostatic hematopoiesis, while vascular ECs secreted PTN under BM ablation. Even though endogenous PTN can regulate hematopoiesis under injury, BM preconditioning involves damage to niche compartments including ECs, as indicated by increased levels of circulating ECs [25]. Therefore, supplementation of MSC CM and/or recombinant PTN may accelerate restoration of BM function. Further studies should investigate the effects of PTN produced from T-MSCs on BM reconstitution and HSC regeneration using loss-of-function studies.

PTN secretion was measured in AT-, BM-, or T-MSC CM and results showed that PTN is detected in a concentration of 83.05 ± 25.53 ng/mL in 10^6^ T-MSCs while it was under the detection limits in AT- or BM-MSCs. The dose of rhPTN injected in the mice was 1 μg/mouse, which is about 10-times more in quantity than PTN dissolved in the CM. Yet, CM treatment showed the effects better than PTN on EC recovery, which suggests that various factors in MSC CM acts in concert to promote angiogenesis. Further studies may involve a development of strategies that can enhance secretion of PTN as well as proangiogenic factors by modulating culture conditions.

The regeneration of ECs is not only shown in BM but also in mesenteric endothelium. By employing ultrastructural analyses of TEM, we observed defects in interendothelial junctions by chemotherapy and their recovery after BMT when supplemented with T-MSC CM or recombinant PTN. Endothelia play important roles in maintaining vascular homeostasis, and intercellular junctions support structural integrity and cell-to-cell communication. High-dose chemotherapy causes significant damage to the endothelium [24,25], and delayed recovery of endothelial integrity may result in defects to vascular function. Regarding HSCT, elevated levels of circulating ECs indicate endothelial damage as well as graft-versus-host disease [41]. Therefore, BMT aided by T-MSC CM or recombinant PTN may promote reconstitution of EC junctions, which would help the migration of transplanted BM cells and protect patients from graft failure.

In summary, we have demonstrated the effectiveness of MSC CM in promoting BM reconstitution after BMT. The efficacy of PTN treatment was examined in parallel and demonstrated that the therapeutic effects of T-MSC CM surpass the effects of PTN for BM EC recovery. These results, to the best of our knowledge, provide the first description of the effects of MSC CM on BM engraftment and BM EC regeneration. Furthermore, these findings highlight the potential therapeutic relevance of T-MSC CM for increasing HSCT efficacy for the cure of hematologic malignancies.

## References

[1] Olsson R.F., Logan B.R., Chaudhury S., Zhu X., Akpek G., Bolwell B.J., Bredeson C.N., Dvorak C.C., Gupta V., Ho V.T. (2015). Primary graft failure after myeloablative allogeneic hematopoietic cell transplantation for hematologic malignancies. Leukemia.

[2] Ratajczak M.Z., Suszynska M. (2016). Emerging Strategies to Enhance Homing and Engraftment of Hematopoietic Stem Cells. Stem Cell Rev..

[3] Wysoczynski M., Reca R., Ratajczak J., Kucia M., Shirvaikar N., Honczarenko M., Mills M., Wanzeck J., Janowska-Wieczorek A., Ratajczak M.Z. (2005). Incorporation of CXCR4 into membrane lipid rafts primes homing-related responses of hematopoietic stem/progenitor cells to an SDF-1 gradient. Blood.

[4] Janowska-Wieczorek A., Majka M., Kijowski J., Baj-Krzyworzeka M., Reca R., Turner A.R., Ratajczak J., Emerson S.G., Kowalska M.A., Ratajczak M.Z. (2001). Platelet-derived microparticles bind to hematopoietic stem/progenitor cells and enhance their engraftment. Blood.

[5] Hoggatt J., Singh P., Sampath J., Pelus L.M. (2009). Prostaglandin E2 enhances hematopoietic stem cell homing, survival, and proliferation. Blood.

[6] Ko K.H., Holmes T., Palladinetti P., Song E., Nordon R., O’Brien T.A., Dolnikov A. (2011). GSK-3beta inhibition promotes engraftment of ex vivo-expanded hematopoietic stem cells and modulates gene expression. Stem Cells.

[7] Capitano M.L., Hangoc G., Cooper S., Broxmeyer H.E. (2015). Mild Heat Treatment Primes Human CD34(+) Cord Blood Cells for Migration Toward SDF-1alpha and Enhances Engraftment in an NSG Mouse Model. Stem Cells.

[8] Mantel C.R., O’Leary H.A., Chitteti B.R., Huang X., Cooper S., Hangoc G., Brustovetsky N., Srour E.F., Lee M.R., Messina-Graham S. (2015). Enhancing Hematopoietic Stem Cell Transplantation Efficacy by Mitigating Oxygen Shock. Cell.

[9] Koc O.N., Gerson S.L., Cooper B.W., Dyhouse S.M., Haynesworth S.E., Caplan A.I., Lazarus H.M. (2000). Rapid hematopoietic recovery after coinfusion of autologous-blood stem cells and culture-expanded marrow mesenchymal stem cells in advanced breast cancer patients receiving high-dose chemotherapy. J. Clin. Oncol.

[10] Ball L.M., Bernardo M.E., Roelofs H., Lankester A., Cometa A., Egeler R.M., Locatelli F., Fibbe W.E. (2007). Cotransplantation of ex vivo expanded mesenchymal stem cells accelerates lymphocyte recovery and may reduce the risk of graft failure in haploidentical hematopoietic stem-cell transplantation. Blood.

[11] Wu Y., Wang Z., Cao Y., Xu L., Li X., Liu P., Yan P., Liu Z., Zhao D., Wang J. (2013). Cotransplantation of haploidentical hematopoietic and umbilical cord mesenchymal stem cells with a myeloablative regimen for refractory/relapsed hematologic malignancy. Ann. Hematol.

[12] Xiong Y.Y., Fan Q., Huang F., Zhang Y., Wang Y., Chen X.Y., Fan Z.P., Zhou H.S., Xiao Y., Xu X.J. (2014). Mesenchymal stem cells versus mesenchymal stem cells combined with cord blood for engraftment failure after autologous hematopoietic stem cell transplantation: A pilot prospective, open-label, randomized trial. Biol. Blood Marrow Transplant..

[13] Liu X., Wu M., Peng Y., Chen X., Sun J., Huang F., Fan Z., Zhou H., Wu X., Yu G. (2014). Improvement in poor graft function after allogeneic hematopoietic stem cell transplantation upon administration of mesenchymal stem cells from third-party donors: A pilot prospective study. Cell Transplant..

[14] Zhao K., Liu Q. (2016). The clinical application of mesenchymal stromal cells in hematopoietic stem cell transplantation. J. Hematol. Oncol..

[15] Park M., Kim Y.H., Ryu J.H., Woo S.Y., Ryu K.H. (2015). Immune suppressive effects of tonsil-derived mesenchymal stem cells on mouse bone-marrow-derived dendritic cells. Stem Cells Int..

[16] Cho K.A., Lee J.K., Kim Y.H., Park M., Woo S.Y., Ryu K.H. (2017). Mesenchymal stem cells ameliorate B-cell-mediated immune responses and increase IL-10-expressing regulatory B cells in an EBI3-dependent manner. Cell Mol. Immunol..

[17] Kim J.Y., Park M., Kim Y.H., Ryu K.H., Lee K.H., Cho K.A., Woo S.Y. (2018). Tonsil-derived mesenchymal stem cells (T-MSCs) prevent Th17-mediated autoimmune response via regulation of the programmed death-1/programmed death ligand-1 (PD-1/PD-L1) pathway. J. Tissue Eng. Regen Med..

[18] Ryu J.H., Park M., Kim B.K., Kim Y.H., Woo S.Y., Ryu K.H. (2016). Human tonsilderived mesenchymal stromal cells enhanced myelopoiesis in a mouse model of allogeneic bone marrow transplantation. Mol. Med. Rep..

[19] Kim Y.H., Park M., Cho K.A., Kim B.K., Ryu J.H., Woo S.Y., Ryu K.H. (2016). Tonsil-Derived Mesenchymal Stem Cells Promote Bone Mineralization and Reduce Marrow and Visceral Adiposity in a Mouse Model of Senile Osteoporosis. Stem Cells Dev..

[20] Ryu K.H., Cho K.A., Park H.S., Kim J.Y., Woo S.Y., Jo I., Choi Y.H., Park Y.M., Jung S.C., Chung S.M. (2012). Tonsil-derived mesenchymal stromal cells: Evaluation of biologic, immunologic and genetic factors for successful banking. Cytotherapy.

[21] Cho K.A., Park M., Kim Y.H., Woo S.Y., Ryu K.H. (2017). RNA sequencing reveals a transcriptomic portrait of human mesenchymal stem cells from bone marrow, adipose tissue, and palatine tonsils. Sci. Rep..

[22] Himburg H.A., Muramoto G.G., Daher P., Meadows S.K., Russell J.L., Doan P., Chi J.T., Salter A.B., Lento W.E., Reya T. (2010). Pleiotrophin regulates the expansion and regeneration of hematopoietic stem cells. Nat. Med..

[23] Himburg H.A., Harris J.R., Ito T., Daher P., Russell J.L., Quarmyne M., Doan P.L., Helms K., Nakamura M., Fixsen E. (2012). Pleiotrophin regulates the retention and self-renewal of hematopoietic stem cells in the bone marrow vascular niche. Cell Rep..

[24] Zeng L., Jia L., Xu S., Yan Z., Ding S., Xu K. (2010). Vascular endothelium changes after conditioning in hematopoietic stem cell transplantation: Role of cyclophosphamide and busulfan. Transplant. Proc..

[25] Al-Hashmi S., Boels P.J., Zadjali F., Sadeghi B., Sallstrom J., Hultenby K., Hassan Z., Arner A., Hassan M. (2012). Busulphan-cyclophosphamide cause endothelial injury, remodeling of resistance arteries and enhanced expression of endothelial nitric oxide synthase. PLoS ONE.

[26] Butler J.M., Nolan D.J., Vertes E.L., Varnum-Finney B., Kobayashi H., Hooper A.T., Seandel M., Shido K., White I.A., Kobayashi M. (2010). Endothelial cells are essential for the self-renewal and repopulation of Notch-dependent hematopoietic stem cells. Cell Stem Cell.

[27] Guo P., Poulos M.G., Palikuqi B., Badwe C.R., Lis R., Kunar B., Ding B.S., Rabbany S.Y., Shido K., Butler J.M. (2017). Endothelial jagged-2 sustains hematopoietic stem and progenitor reconstitution after myelosuppression. J. Clin. Invest..

[28] Kusumbe A.P., Ramasamy S.K., Itkin T., Mae M.A., Langen U.H., Betsholtz C., Lapidot T., Adams R.H. (2016). Age-dependent modulation of vascular niches for haematopoietic stem cells. Nature.

[29] Kusumbe A.P., Ramasamy S.K., Adams R.H. (2014). Coupling of angiogenesis and osteogenesis by a specific vessel subtype in bone. Nature.

[30] Fernandez-Garcia M., Yanez R.M., Sanchez-Dominguez R., Hernando-Rodriguez M., Peces-Barba M., Herrera G., O’Connor J.E., Segovia J.C., Bueren J.A., Lamana M.L. (2015). Mesenchymal stromal cells enhance the engraftment of hematopoietic stem cells in an autologous mouse transplantation model. Stem Cell Res. Ther..

[31] Park M., Kim Y.H., Woo S.Y., Lee H.J., Yu Y., Kim H.S., Park Y.S., Jo I., Park J.W., Jung S.C. (2015). Tonsil-derived mesenchymal stem cells ameliorate CCl4-induced liver fibrosis in mice via autophagy activation. Sci. Rep..

[32] Vizoso F.J., Eiro N., Cid S., Schneider J., Perez-Fernandez R. (2017). Mesenchymal Stem Cell Secretome: Toward Cell-Free Therapeutic Strategies in Regenerative Medicine. Int. J. Mol. Sci..

[33] Harrell C.R., Fellabaum C., Jovicic N., Djonov V., Arsenijevic N., Volarevic V. (2019). Molecular Mechanisms Responsible for Therapeutic Potential of Mesenchymal Stem Cell-Derived Secretome. Cells.

[34] Bhang S.H., Lee S., Shin J.Y., Lee T.J., Jang H.K., Kim B.S. (2014). Efficacious and clinically relevant conditioned medium of human adipose-derived stem cells for therapeutic angiogenesis. Mol. Ther..

[35] Cho K.A., Lee H.J., Jeong H., Kim M., Jung S.Y., Park H.S., Ryu K.H., Lee S.J., Jeong B., Lee H. (2019). Tonsil-derived stem cells as a new source of adult stem cells. World J. Stem Cells.

[36] Oh S.Y., Choi Y.M., Kim H.Y., Park Y.S., Jung S.C., Park J.W., Woo S.Y., Ryu K.H., Kim H.S., Jo I. (2019). Application of Tonsil-Derived Mesenchymal Stem Cells in Tissue Regeneration: Concise Review. Stem Cells.

[37] Sahin A.O., Buitenhuis M. (2012). Molecular mechanisms underlying adhesion and migration of hematopoietic stem cells. Cell Adh. Migr..

[38] Zheng Y., Watanabe N., Nagamura-Inoue T., Igura K., Nagayama H., Tojo A., Tanosaki R., Takaue Y., Okamoto S., Takahashi T.A. (2003). Ex vivo manipulation of umbilical cord blood-derived hematopoietic stem/progenitor cells with recombinant human stem cell factor can up-regulate levels of homing-essential molecules to increase their transmigratory potential. Exp. Hematol..

[39] Vagima Y., Avigdor A., Goichberg P., Shivtiel S., Tesio M., Kalinkovich A., Golan K., Dar A., Kollet O., Petit I. (2009). MT1-MMP and RECK are involved in human CD34+ progenitor cell retention, egress, and mobilization. J. Clin. Invest..

[40] Himburg H.A., Termini C.M., Schlussel L., Kan J., Li M., Zhao L., Fang T., Sasine J.P., Chang V.Y., Chute J.P. (2018). Distinct Bone Marrow Sources of Pleiotrophin Control Hematopoietic Stem Cell Maintenance and Regeneration. Cell Stem Cell.

[41] Zeng L., Yan Z., Ding S., Xu K., Wang L. (2008). Endothelial injury, an intriguing effect of methotrexate and cyclophosphamide during hematopoietic stem cell transplantation in mice. Transplant. Proc..

